# Assessment of measurable residual disease in ovarian tissue collected for fertility preservation in patients in remission from acute myeloid leukaemia: A pilot study

**DOI:** 10.1111/bjh.70289

**Published:** 2025-12-19

**Authors:** Augustin Boudry, Florian Chevillon, Alice Marceau‐Renaut, Thorsten Braun, Thomas Boyer, Nathalie Helevaut, Elise Fournier, Sandrine Geffroy, Nicolas Boissel, Emmanuelle Clappier, Claude Preudhomme, Nicolas Duployez, Catherine Poirot, Laurène Fenwarth

**Affiliations:** ^1^ Laboratory of Hematology CHU Lille Lille France; ^2^ Université de Lille, CHU de Lille, ULR 2694—METRICS Lille France; ^3^ Hematology Adolescents and Young Adult Unit Saint‐Louis Hospital, Assistance Publique‐Hôpitaux de Paris Paris France; ^4^ Cancer Heterogeneity Plasticity and Resistance to Therapies (CANTHER), UMR9020‐U1277 University of Lille Lille France; ^5^ Department of Hematology Université Paris Nord, Hôpital Avicenne, AP‐HP Bobigny France; ^6^ Research Unit EA‐3518, Institut de Recherche Saint‐Louis Université de Paris Paris France; ^7^ Laboratory of Hematology CHU Amiens‐Picardie Amiens France; ^8^ Laboratory of Hematology Saint Louis Hospital, Assistance Publique‐Hôpitaux de Paris (AP‐HP) Paris France

**Keywords:** acute myeloid leukaemia, fertility preservation, measurable residual disease, ovarian tissue cryopreservation

## Abstract

Allogeneic haematopoietic stem cell transplantation (ASCT) is a curative treatment for acute myeloid leukaemia (AML) but carries a high risk of gonadotoxicity. Ovarian tissue cryopreservation (OTC) offers a fertility preservation option, yet its safety in AML remains uncertain due to the risk of leukaemic cell reintroduction. The FERTILAM pilot study evaluated measurable residual disease (MRD) in ovarian tissue collected at complete remission (CR) from nine AML patients undergoing OTC before ASCT. MRD was assessed using patient‐specific clonal markers via droplet digital polymerase chain reaction on DNA and RNA from bone marrow (BM), ovarian cortex and medulla. At CR, MRD‐DNA was detected in ovarian cortex of four of nine patients, all with concurrent MRD positivity in BM. Three patients were negative in both BM and ovarian tissue. Paired cortex/medulla analyses showed concordant MRD‐DNA results in five of six patients. BM MRD‐RNA and MRD‐DNA were fully concordant, whereas two discrepancies were observed between MRD‐DNA and MRD‐RNA in ovarian tissue. These findings suggest potential leukaemic cell persistence in ovarian tissue despite CR and highlight the need for sensitive molecular assays to assess safety prior to ovarian tissue transplantation.

## INTRODUCTION

Allogeneic haematopoietic stem cell transplantation (ASCT) remains the cornerstone of curative treatment for acute myeloid leukaemia (AML), particularly in patients with adverse prognostic markers or relapsed disease. While ASCT has significantly improved survival outcomes, especially among adolescents and young adults, the use of myeloablative conditioning regimens carries a high risk of gonadotoxicity. This toxicity often results in premature ovarian insufficiency, impairing both ovarian endocrine function and fertility potential.^1^, ^2^ As survival outcomes continue to improve, long‐term survivorship issues—particularly fertility preservation—have become important considerations in patient care.

Ovarian tissue cryopreservation (OTC) represents a relevant fertility preservation strategy in female patients requiring highly gonadotoxic treatment for cancers.^3^ Ovarian tissue transplantation (OTT) has emerged as an effective technique, with reported live birth rates ranging from 19% to 32%, resulting in more than 200 live births to date.^4^, ^5^, ^6^ Beyond fertility restoration, OTT has also demonstrated high efficacy in re‐establishing ovarian endocrine function, with endocrine restoration reported in 70%–95% of women undergoing the procedure.^6^

In acute leukaemia, the autograft of cryopreserved ovarian cortex for fertility purposes or to restore endocrine function may pose the risk of reintroducing residual leukaemic cells. Importantly, recent evidence suggests that the risk of leukaemic cell reintroduction through OTT is substantially reduced in patients who are measurable residual disease (MRD)‐negative in bone marrow (BM) at the time of ovarian tissue collection, as shown in a recent analysis of leukaemia patients undergoing OTC.^7^ In acute lymphoblastic leukaemia, MRD‐based technologies identified leukaemic cells in ovarian fragments.^8^ Such data are currently lacking in AML patients. Previous reports highlighted the limited sensitivity of histology and immunochemistry compared to molecular MRD approaches.^9^ Here, we aimed to assess molecular MRD in ovarian tissue collected at the time of complete remission (CR) in AML patients.

## MATERIALS AND METHODS

### Study population

The FERTILAM study (ClinicalTrials.gov ID #NCT04679285) is a pilot descriptive study evaluating sensitive molecular backtracking of leukaemic cells in cryopreserved ovarian fragments collected at CR in young patients intensively treated for AML. CR was defined as <5% BM blasts, normal peripheral counts and absence of extramedullary disease. After an ovarian pickup, the ovarian cortex was separated from the medulla, cut into fragments and cryopreserved using a slow‐freezing method.^10^ Only patients with at least 15 cortical fragments were included in this study to ensure the possibility of subsequent reimplantation of ovarian cortex fragments. For each patient, only one cortical fragment and one medullary fragment were allocated to the MRD assessment. Similarly, when available, the medulla was preserved using the same method. This study was approved by the CPP OUEST II and was done in accordance with the Declaration of Helsinki.

A total of nine AML patients aged up to 40 years who underwent OTC before ASCT were included.

### Molecular analysis at diagnosis

At diagnosis, comprehensive molecular screening was conducted on BM samples to detect both mutations and fusion genes. Mutations were identified on genomic DNA using a capture‐based next‐generation sequencing (NGS) custom panel targeting 90 genes (additional information available in Supporting Information S1). Fusion genes were initially screened by reverse transcription‐multiplex ligation probe amplification.^11^ Genomic confirmation was performed by breakpoint analysis, leveraging soft‐clipped reads identified by a custom‐designed targeted NGS panel. This panel covered both intronic and exonic regions of 31 full‐length genes implicated in myeloid rearrangements (Table S1). Sequencing was carried out on the Illumina® NovaSeq 6000 platform, using paired‐end sequencing (2 × 151 bp). The regions of interest were sequenced at an average depth of 3000× for the single nucleotide variants (SNVs) panel and 200× for the structural variants (SVs) panel. The bioinformatics workflow implemented in this study adhered to a systematic protocol. BCL files generated by the sequencer were first converted into FASTQ format using BCL Convert (v3.8.4). The resulting FASTQ files were subsequently quality‐trimmed with fastp (v0.20.0). Processed reads were then aligned to the human reference genome (hg19) using the BWA aligner (v0.7.17). SNVs were detected using two independent variant callers: Mutect2 (v4.1.0.0) and VarDict (v1.5.8), with subsequent annotation performed using the Variant Effect Predictor (v92.1). SVs were identified using Manta (v1.6.0) and GRIDSS (v2.13.2), and annotated with AnnotSV (v3.2.2). This integrated analytical strategy ensured a comprehensive characterization of founder alterations across the targeted genomic regions (Figure S1).

### Ovarian tissue preparation and MRD evaluation

Ovarian tissue slices were thawed at 4°C. A quarter to a half of the slices were selected for the analysis. The tissue fragments were weighed, then finely dissected. These fragments were placed in a 1.5 mL tube with 500 × 10^−6^ L of phosphate‐buffered saline (PBS) and vortexed for uniform mixing. After centrifugation at 350 *g* for 7 min at 4°C, the supernatants were discarded, leaving the prepared cortex and medulla tissues ready for nucleic acid extraction. The DNA and RNA were then extracted manually using the NucleoSpin TISSUE DNA kit® and the NucleoSpin TISSUE RNA kit® from Macherey‐Nagel, respectively, in accordance with the manufacturer's instructions.

**FIGURE 1:**
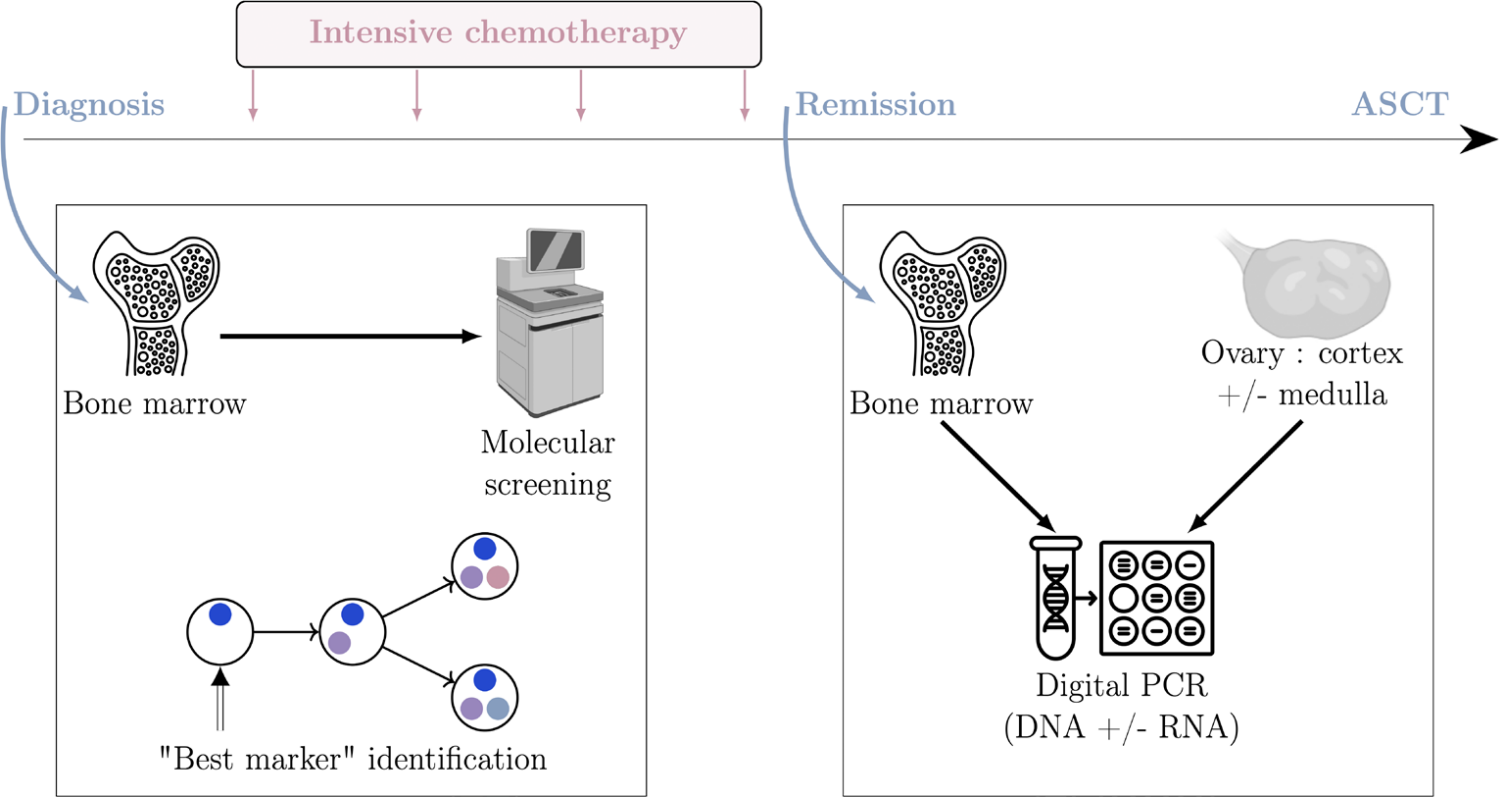
Workflow of ovarian measurable residual disease (MRD) assessment in acute myeloid leukaemia (AML) patients during complete remission. The leukaemia‐specific MRD marker was first identified in the bone marrow at AML diagnosis by next‐generation sequencing analysis. After achieving complete remission, ovarian tissue (cortex ± medulla) was collected for cryopreservation. MRD markers identified at diagnosis were assessed in bone marrow at remission and in ovarian tissue (cortex ± medulla) by digital PCR. ASCT, allogeneic haematopoietic stem cell transplantation.

Clonal markers were tracked on BM and ovarian samples (cortex and medulla when available) collected at CR, using tailored primer and probe designs for droplet digital polymerase chain reaction (ddPCR) analysis (Figure 1). While complementary DNA transcribed from RNA serves as the preferred template in haematopoietic tissues,^12^, ^13^ the impact of the ovarian microenvironment on the transcriptome remains unknown. Thus, we adopted a DNA‐based approach for the primary assessment of ovarian MRD. Whenever feasible, molecular markers were analysed both at genomic (MRD‐DNA) and transcript (MRD‐RNA) levels, with a sensitivity threshold ranging from 10^−3^ to 10^−5^ depending on the molecular marker (additional information available in Tables S2 and S3). Mutation quantification was reported as a fractional abundance (FA: mutated allele divided by total allele), except for *UBTF*. The quantification of the *UBTF* mutation was reported as a ratio of the mutation relative to the *ALB* gene due to the high homology between the *UBTF* mutation and the reference sequence. For the different SNVs detection systems, the mutated alleles and the wild‐type alleles/*ALB* gene alleles were identified using probes labelled with FAM and HEX fluorophores, respectively. Fusion quantification was reported as a ratio of the fusion (labelled FAM) relative to the housekeeping gene (*ABL1* on cDNA/*ALB* on gDNA, labelled HEX).

MRD evaluation by multiparametric flow cytometry (MFC), which requires fresh tissue, was feasible in a single patient. The cortex and medulla tissues were processed together. Briefly, the ovarian biopsy was dissociated by the GentleMACS dissociator (Miltenyi Biotec) using the appropriate program. After filtration and counting, in order to adjust the suspension to 500 × 10^5^ cells, direct labelling was performed with the following antibodies: anti‐CD7‐FITC (clone 8H8.1, Iotest, Beckman Coulter), anti‐CD13‐PE (clone SJ1D1, Iotest, Beckman Coulter), anti‐HLA‐DR‐ECD (clone Immu‐357, Iotest, Beckman Coulter), anti‐CD33‐PC5.5 (clone D3HL60.251, Iotest, Beckman Coulter), anti‐CD38‐PE‐Cy7 (clone LS198.4.3, Iotest, Beckman Coulter), anti‐CD34‐APC (clone 581, Iotest, Beckman Coulter), anti‐CD56‐Alexa Fluor 700 (clone N901, Iotest, Beckman Coulter), anti‐CD19‐Alexa Fluor 750 (clone J3‐119, Iotest, Beckman Coulter), anti‐CD117‐BV421 (clone 104D2, Beckton Dickinson) and anti‐CD45‐Krom Orange (clone J33, Iotest, Beckman Coulter). After 20 min of incubation at room temperature, the samples were washed once in PBS + 2% fetal bovine serum then resuspended in 400 × 10^−6^ L of PBS. Data acquisition was performed on a Navios flow cytometer and analysed with Kaluza software (Beckman Coulter).

## RESULTS

Among the nine AML patients included, the median age at diagnosis was 24 years (range: 10–34), and the median interval between achievement of CR and OTC was 13 days (range: 3–60). Six patients had a fusion gene (#2, #3, #4, #6, #7, #8) and three had at least one driver clonal mutation (#1, #5, #9) (Table 1; Table S4). This repartition was in line with the high prevalence of fusions in AML in children and younger adults.^14^ CR was achieved after induction in all patients except patient #5, who achieved CR following two additional lines of treatment. Of note, patients #3, #8 and #9 experienced relapse prior to OTC. The median duration of follow‐up after ASCT was 3.8 years. Two patients (#5 and #7) experienced relapse following ASCT (Table S4). At the time of OTC, three patients had undetectable MRD‐DNA in both BM and ovarian cortex (#1, #6, #8), while four had positive MRD‐DNA in both BM and ovarian cortex (#3, #5, #7, #9). Two patients (#2 and #4) had positive MRD‐DNA in the BM and undetectable MRD‐DNA in the ovarian cortex. A comparison was conducted between ovarian cortex and medulla for six patients. This revealed complete concordance in five patients, whereas the final patient (#2) exhibited positive MRD‐DNA in the ovarian medulla but undetectable MRD‐DNA in the ovarian cortex (Table 1). In the entire set of BM samples analysed, there was complete concordance between MRD‐DNA and MRD‐RNA. A comparison between MRD‐DNA and MRD‐RNA was conducted in 10 ovarian tissues (cortex, *n* = 6; medulla, *n* = 4) from six patients. Only two discrepancies were noted, with MRD‐DNA detected as positive while MRD‐RNA remained undetectable (in the ovarian cortex of patient #3 and in the ovarian medulla of patient #2).

**TABLE 1:** Measurable residual disease (MRD) results in ovarian samples and bone marrow.

No.	Age at AML diagnosis	AML subtype*	Molecular marker(s) for MRD	Bone marrow MRD‐DNA	Ovarian cortex; MRD‐DNA	Ovarian medulla; MRD‐DNA	Bone marrow MRD‐RNA	Ovarian cortex; MRD‐RNA	Ovarian medulla MRD‐RNA	Follow‐up after ASCT (years)	Relapse post‐ASCT	Time from ACST to relapse (years)
1	21	Myelodysplasia‐related	*RUNX1* *PHF6*	UD	UD	NA	NA	NA	NA	10.5	N	—
2	34	*DEK::NUP214* fusion	*DEK::NUP214*	2 × 10^−4^	UD	Positive <10^−4^	1 × 10^−3^	UD	UD	8.5	N	—
3	28	*CBFB::MYH11* fusion	*CBFB::MYH11*	Positive <10^−4^	4 × 10^−4^	4 × 10^−4^	Positive <10^−4^	UD	Positive <10^−4^	3.3	N	—
4	23	*KMT2A* rearrangement	*KMT2A::MLLT3*	Positive <10^−4^	UD	NA	NA	NA	NA	11.0	N	—
5	25	Other defined genetic alterations: *UBTF* mutation	*UBTF*	2 × 10^−1^	Positive <10^−4^	5 × 10^−4^	NA	NA	NA	3.8	Y	3.4
6	17	*KMT2A* rearrangement	*KMT2A::MLLT10*	UD	UD	UD	UD	UD	NA	3.2	N	—
7	28	Other defined genetic alterations: *ETV6::SYK* fusion	*ETV6::SYK*	5 × 10^−2^	2 × 10^−3^	3 × 10^−3^	3 × 10^−1^	1 × 10^−3^	2 × 10^−3^	0.8	Y	0.6
8	24	*RUNX1::RUNX1T1* fusion	*RUNX1::RUNXT1*	UD	UD	UD	UD	UD	UD	2.7	N	—
9	10	*NPM1* mutation	*NPM1*	7 × 10^−4^	6 × 10^−4^	NA	Positive <10^−4^	1 × 10^−3^	NA	5.2	N	—

Abbreviations: AML, acute myeloid leukaemia; ASCT, autologous stem cell transplantation; MRD‐DNA, measurable residual disease on DNA; MRD‐RNA, measurable residual disease on RNA; N, no; NA, not available; UD, undetectable; Y, yes.

* According to the WHO 2022 classification.^15^

**FIGURE 2:**
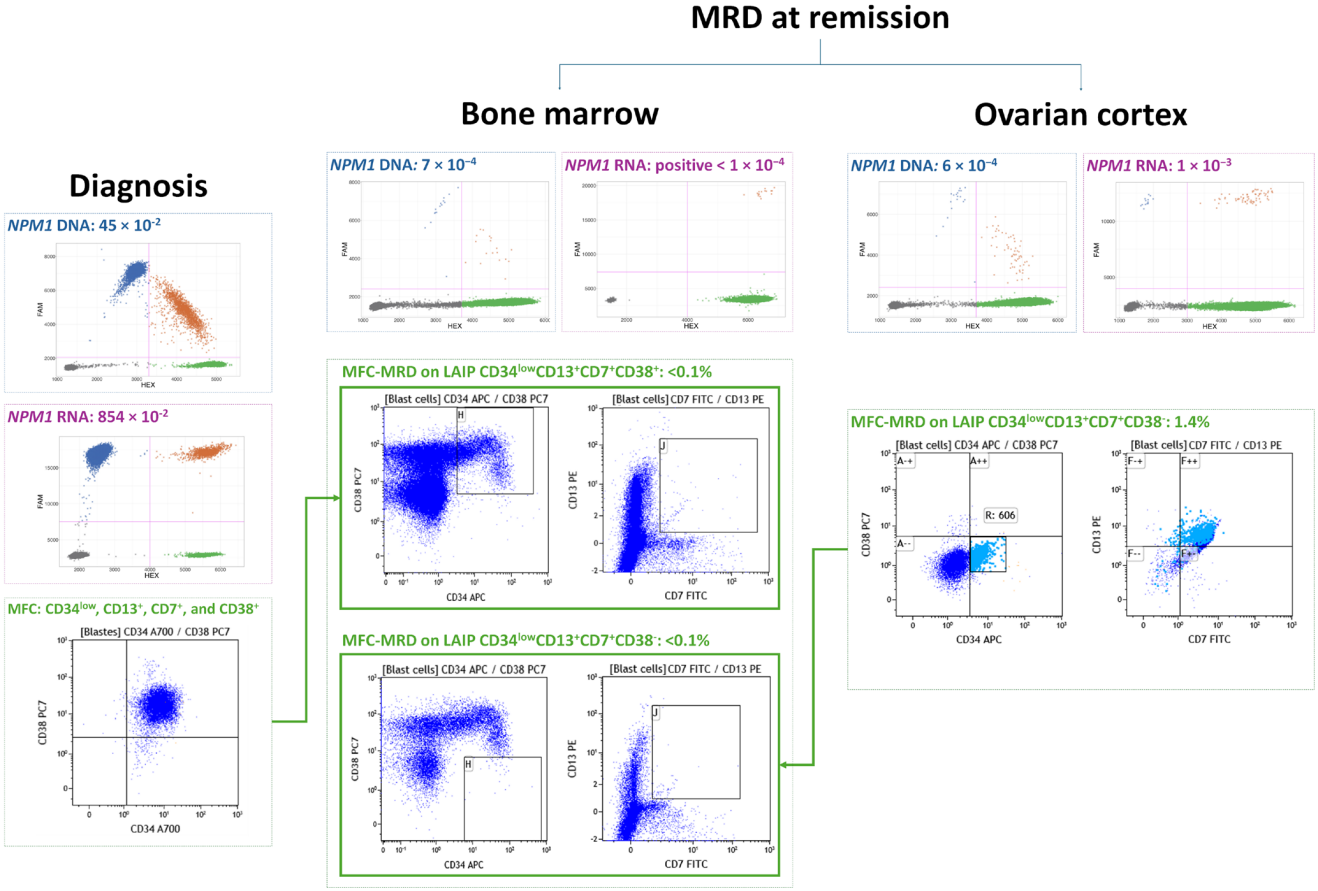
Comparison of droplet digital PCR (ddPCR) measurable residual disease (MRD) and multiparametric flow cytometry (MFC) MRD in patient #9. ddPCR MRD: 2D fluorescence scatter plots displaying duplicate wells of a mixed *NPM1*‐positive versus wild‐type sample on the DNA matrix (blue boxes) and *NPM1*‐positive versus *ABL1* copies on the RNA matrix (purple boxes). For each scatter plot, the grey cluster represents negative droplets, the green cluster represents droplets positive for wild‐type (DNA)/*ABL1* copies (RNA), the blue cluster represents droplets positive for *NPM1* mutation only, the orange cluster represents droplets positive for both *NPM1* mutation and wild‐type (DNA)/*ABL1* targets (RNA). MFC‐MRD: at remission, MFC‐MRD on bone marrow was negative for both the LAIP phenotype CD34^low^CD13^+^CD7^+^CD38^+^ detected in the bone marrow at diagnosis and the LAIP phenotype CD34^low^CD13^+^CD7^+^CD38^−^ identified in the ovarian cortex at remission.

The MFC analysis performed on the BM at AML diagnosis in patient #9 revealed the following leukemia‐associated immunophenotype (LAIP) population: CD34^low^, CD13^+^, CD33^+^, CD7^+^ and CD38^+^. The MFC‐MRD assessed at the time of ovarian sampling was undetectable (<0.1%) in the BM but revealed a population expressing CD34^low^, CD13^+^, CD33^+^, CD7^+^, but CD38^−^ at a level of 1.4% in the ovarian cortex. Concurrent analysis of *NPM1* MRD (on DNA and RNA) was positive in the BM (*NPM1* MRD‐DNA: 7 × 10^−4^; *NPM1* MRD‐RNA: positive <1 × 10^−4^) and in the ovarian cortex (*NPM1* MRD‐DNA: 6 × 10^−4^; *NPM1* MRD‐RNA: 1 × 10^−3^) (Figure 2).

## DISCUSSION

Molecular backtracking found tumour DNA in four of nine patients' ovarian cortex, aligning with positive BM MRD, while three patients with undetectable BM MRD were also negative for ovarian MRD. This pilot study is the first report highlighting the presence of myeloid blasts in ovarian tissue by molecular approaches with a high sensitivity (10^−3^ to 10^−5^). Although the number of patients included was low (*n* = 9 patients), these results suggest that ovarian tissue may serve as a reservoir for leukaemic cells. All patients received intensive treatment regimens; however, chemotherapy regimens, including drug combinations and dosing, were not homogeneous. Furthermore, AML subtypes and prognostic profiles varied across the cohort. This heterogeneity in treatment exposure and disease characteristics may have influenced MRD outcomes, representing a relative limitation of the study. MRD‐DNA discrepancies observed between BM and ovarian cortex in patients #2 and #4 should be interpreted with caution. In this feasibility study, a single fragment of ovarian cortex was analysed. Undetectable MRD‐DNA in the ovarian cortex, while BM MRD‐DNA remains positive, does not preclude the possibility of tumoral heterogeneity within the ovarian cortex. Assessing at least two fragments and applying complementary techniques could enhance MRD detection sensitivity and reliability, as illustrated by recent case reports of live births following OTT in AML survivors.^16^, ^17^, ^18^ MRD‐DNA on cortex and medulla was similar for all patients except patient #3. However, the ovarian medulla is a highly vascular structure. Regarding patient #3, the positive MDR‐DNA in the ovarian medulla may reflect haematopoietic contamination. A major limit of such a study remains the threshold for leukaemic cells detection despite the high sensitivity of the ddPCR technology. Undetectable MRD in the ovarian cortex does not exclude the presence of remaining blast cells.

The concordance rate between MRD‐DNA and MRD‐RNA on BM samples was 100% (*n* = 6 patients). The comparison between MRD‐DNA and MRD‐RNA on ovarian tissue could be performed for six patients: four showed a concordance between the two matrices, while the last patients (#2, #3) had positive ovarian MRD only on genomic DNA (*DEK::NUP214* ratio 4.7 × 10^−5^ and *CBFB::MYH11* ratio 4 × 10^−4^). Discrepancies between MRD‐DNA and MRD‐RNA likely reflect the inherent instability of RNA, which is prone to degradation during sample handling and can compromise assay sensitivity, potentially resulting in false‐negative results, whereas MRD‐DNA assays remain comparatively robust. For *DEK::NUP214* fusion, this discrepancy could also be attributed to the difference in limits of detection between MRD‐DNA (10^−5^) and MRD‐RNA (10^−4^). However, these differences might also reflect the lack of expression of the fusion genes by the leukaemic cells in the ovarian tissue. Since *DEK::NUP214* and *CBFB::MYH11* fusion transcripts are typically highly expressed by leukaemic cells in the BM,^13^ it is possible that the ovarian microenvironment restricts their expression. Nonetheless, this hypothesis needs to be validated in a larger cohort. Similar discrepancies were observed in a previous study investigating molecular MRD in ovarian cortex and medulla in patients treated for acute lymphoblastic leukaemia.^8^

MRD is a well‐recognized post‐treatment prognostic factor in AML, yet it remains a major challenge, requiring highly sensitive technologies to detect residual leukaemic cells and guide therapeutic decisions. MRD can be monitored by MFC, quantitative PCR (qPCR)‐based assays targeting recurrent mutations or fusion transcripts, or by NGS‐based assays, as recommended by the European LeukemiaNet (ELN) consensus.^13^ MFC identifies an LAIP combination in ≈90% of AML cases at diagnosis.^19^ Each LAIP may have its own background signal, which can influence both the sensitivity and the limit of quantification of MFC‐MRD. The detection threshold of MFC‐MRD ranges from 10^−3^ to 10^−4^ but may be affected by inter‐operator variability despite standardized panels. Additionally, MFC‐MRD requires fresh samples. Molecular MRD assessment, including qPCR, ddPCR and NGS, allows detection of RNA/DNA molecular targets (10^−3^ to 10^−5^). qPCR remains the most validated method but applies to only 40%–60% of AML cases, whereas ddPCR and NGS can assess a broader range of molecular targets. In the current study, most patients were included and screened retrospectively, precluding MRD assessment by MFC. Under these conditions, ddPCR was therefore selected as the method for MRD monitoring. This approach also reflects real‐life scenarios in which MRD evaluation on ovarian tissue may be requested long after OTC, in the context of OTT to restore endocrine function or for reproductive purposes. Compared with MFC, ddPCR offers a more standardized and potentially more reproducible approach, with greater precision in quantification and the added advantage of being applicable to stored or frozen samples, although it requires defined molecular markers. Differences in the biological nature of the markers assessed and in the sensitivity of each assay may account for occasional discrepancies between MFC‐ and molecular‐based MRD results. Combining both approaches yields complementary insights for a more accurate evaluation of leukaemic contamination in ovarian samples. MRD testing by MFC was available in a single patient (#9). However, the LAIP approach revealed a distinct population in the ovarian cortex compared to the BM, with a loss of CD38 expression on the LAIP. This suggests that the ovarian microenvironment may influence the pattern of LAIP expression.

Fertility preservation represents a critical and time‐sensitive component of the comprehensive oncologic management of young patients and should be systematically addressed at the earliest stages of treatment planning, in accordance with the current clinical guidelines.^6^ As the demand for ovarian cortex transplantation to restore fertility or endocrine function increases, highly sensitive tumour genome detection techniques are essential to guide clinical decision‐making, given the risk of disease reintroduction despite the proven efficacy of the procedure. In this context, the strong concordance between MRD‐DNA levels in BM and ovarian cortex suggests that detectable MRD in the BM may reflect a concomitant leukaemic contamination in the ovarian, although the limited data preclude firm conclusions.^7^, ^20^, ^21^, ^22^, ^23^

Several case reports have documented the successful restoration of ovarian function and the achievement of live births in patients with AML when the OTC was performed during CR.^5^, ^16^, ^18^, ^22^ Notably, no cases of AML relapse were reported following OTT, suggesting that, under specific conditions, the risk of disease reintroduction via the graft may be low. Dolmans et al. conducted experiments using xenografts with ovarian tissue from acute lymphoblastic leukaemia or chronic myeloid leukaemia patients in SCID mice and showed the viability and malignancy of leukaemic cells from human ovarian samples taken at diagnosis.^23^ The potential of residual leukaemic cells to cause relapse remains a concern. Experimental models of artificial ovary and xenograft transplantation suggest that small numbers of residual leukaemic cells may be insufficient to trigger relapse, although the risk is higher in patients who are MRD‐positive at the time of OTC compared to MRD‐negative patients.^7^, ^20^, ^22^ Xenotransplantation of ovarian tissue into immunodeficient mice can help to evaluate OTT safety, but it does not capture the potential allogeneic graft‐versus‐leukaemia effect, which may counteract leukaemia development from a low tumour burden. MRD detected in ovarian tissue could potentially be eliminated after reimplantation into a patient with a newly reconstituted immune system following BM transplantation, as previously suggested by Sönmezer et al.^22^ Therefore, current guidelines recommend assessing MRD in ovarian tissue prior to transplantation in order to reduce the potential risk of disease reintroduction.^6^, ^24^

Based on our findings in AML, we observed slight discrepancies in MRD results between the ovarian cortex, the ovarian medulla and the BM. This pilot study suggests that positive BM‐MRD is likely associated with ovarian leukaemic contamination. Indeed, the ovarian cortex is precious since it is the essential component for restoring fertility. However, it is crucial to further confirm the concordance rates across distinct sites and to combine MRD techniques in larger cohorts.

## AUTHOR CONTRIBUTIONS

LF, ND, NB and CPr conceived and planned the experiments. AB, ND, TBo and LF wrote the manuscript. FC, TBr, CPo and EC managed patients and provided clinical and biological data. AB, AM‐R, EF, TBo, SG and NH performed experimentation and developed tools for analysis. AB, LF, ND, AM and EF interpreted molecular data.

## CONFLICT OF INTEREST STATEMENT

The authors declare no conflicts of interest.

## Supporting information


Data S1.

## Data Availability

The data that support the findings of this study are available from the corresponding author upon reasonable request.
