# Supplementary material for: Assessment of measurable residual disease in ovarian tissue collected for fertility preservation in patients in remission from acute myeloid leukaemia: A pilot study
Source: Br J Haematol. 2025 Dec 19;208(3):897–904. doi: 10.1111/bjh.70289 (PMC12995536; doi:10.1111/bjh.70289)
Supplement: Supplementary file 1 — Data S1. [file BJH-208-897-s001.docx]

Supplementary Material

**Assessment of measurable residual disease in ovarian tissue collected for fertility preservation in patients in remission from acute myeloid leukaemia: a pilot study**

Augustin Boudry, Florian Chevillon, A. Marceau-Renaut *and al*.

[Supplementary Material 2](#_Toc213315686)

[High throughput sequencing 2](#_Toc213315687)

[Supplementary Figures 3](#_Toc213315688)

[Figure S1: Bioinformatics pipeline 3](#_Toc213315689)

[Supplementary Tables 4](#_Toc213315690)

[Table S1: Targets of SV panel (Hg19) 4](#_Toc213315691)

[Table S2: ddPCR assay parameters for DNA-based SNVs and fusion genes 5](#_Toc213315692)

[Table S3: ddPCR assay parameters for RNA-based mutation and fusion transcripts 5](#_Toc213315693)

[Table S4: Treatment received prior to OTC and disease evalutation after treatment 6](#_Toc213315694)

# Supplementary Material

## High throughput sequencing

For the identification of single nucleotide variants (SNV), libraries were prepared with a capture-based approach (Twist Bioscience®), using the following custom-designed 90-gene panel:

*ABL1* (NM_005157.4; e04-09), *ANKRD26* (NM_014915.2; 5'UTR), *ASXL1* (NM_015338.5; e11-12), *ASXL2* (NM_018263.4; e11-12), *ATRX* (NM_000489.4; e01-35), *BCOR* (NM_001123385.1; e02-15), *BCORL1* (NM_021946.4; e01-12), *BRAF* (NM_004333.4; e11,15), *CALR* (NM_004343.3; e09), *CBL* (NM_005188.3; e08-09), *CEBPA* (NM_004364.4; e01), *CHEK2* (NM_001005735.1; e02-15), *CREBBP* (NM_004380.2; e01-31), *CRLF2* (NM_022148.3; e06), *CSF3R* (NM_156039.3; e14-17), *CUX1* (NM_181552.3; e01-24), *DDX41* (NM_016222.3; e01-17), *DHX15* (NM_001358.2; e03), *DHX34* (NM_014681.5; e02-17), *DNMT3A* (NM_022552.4; e02-23), *EP300* (NM_001429.3; e01-31), *ETNK1* (NM_018638.4; e01-08), *ETV6* (NM_001987.4; e01-08), *EZH2* (NM_004456.4; e02-20), *FBXW7* (NM_033632.3; e02-12), *FGFR1* (NM_001174067.1; e02-19), *FLT3* (NM_004119.2; e01-24), *GATA1* (NM_002049.3; e02-03), *GATA2* (NM_032638.4; e02-06,c.1017+532_+699), *GNAS* (NM_000516.5; e08-09), *GNB1* (NM_002074.4; e05-06), *HRAS* (NM_005343.3; e02-04), *IDH1* (NM_005896.3; e03-10), *IDH2* (NM_002168.3; e01-11), *IKZF1* (NM_006060.5; e02-08), *IL7R* (NM_002185.3; e06), *JAK1* (NM_002227.3; e02-25), *JAK2* (NM_004972.3; e03-25), *JAK3* (NM_000215.3; e02-24), *KDM6A* (NM_001291415.1; e01-30), *KIT* (NM_000222.2; e08-11,17), *KMT2A* (NM_001197104.1; e01-36), *KRAS* (NM_033360.3; e02-04), *LUC7L2* (NM_001244585.1; e01-11), *MBD4* (NM_003925.2; e01-08), *MPL* (NM_005373.2; e01-12), *MYC* (NM_002467.4; e01-03), *NF1* (NM_001042492.2; e01-58), *NFE2* (NM_001136023.2; e02-03), *NIPBL* (NM_133433.3; e02-47), *NOTCH1* (NM_017617.4; e01-34), *NPM1* (NM_002520.6; e10-11), *NRAS* (NM_002524.4; e02-04), *PAX5* (NM_016734.2; e01-10), *PDGFRA* (NM_006206.5; e02-23), *PDGFRB* (NM_002609.3; e02-23), *PHF6* (NM_001015877.1; e02-10), *PIGA* (NM_002641.3; e02-06), *PPM1D* (NM_003620.3; e01-06), *PRPF8* (NM_006445.3; e02-43), *PTEN* (NM_000314.6; e01-09), *PTPN11* (NM_002834.4; e01-15), *RAD21* (NM_006265.2; e02-14), *RIT1* (NM_006912.5; e05), *RUNX1* (NM_001754.4; e02-09), *SAMD9* (NM_017654.3; e03), *SAMD9L* (NM_152703.3; e05), *SETBP1* (NM_015559.2; e02-06), *SETD2* (NM_014159.6; e01-21), *SF3B1* (NM_012433.3; e13-16), *SH2B3* (NM_005475.2; e02-08), *SMC1A* (NM_006306.3; e01-25), *SMC3* (NM_005445.3; e01-29), *SRP72* (NM_006947.3; e01-19), *SRSF2* (NM_003016.4; e01), *STAG2* (NM_001042749.2; e03-35), *STAT3* (NM_003150.3; e02-24), *STAT5A* (NM_003152.3; e03-20), *STAT5B* (NM_012448.3; e02-19), *TERC* (NR_001566.1; e01), *TERT* (NM_198253.2; e01-16), *TET2* (NM_001127208.2; e03-11), *TP53* (NM_001126112.2; e02-11), *TYK2* (NM_003331.4; e03-25), *U2AF1* (NM_006758.2; e02,06), *UBA1* (NM_003334.3; e03), *UBTF* (NM_014233;3; e10-15), *WT1* (NM_024426.4; e01-10), *ZBTB7A* (NM_015898.3; e02-03), *ZRSR2* (NM_005089.3; e01-11)

# Supplementary Figures

## Figure S1: Bioinformatics pipeline

# Supplementary Tables

## Table S1: Targets of SV panel (Hg19)

| Gene | Chromosome | Start | Stop | Transcript |
| --- | --- | --- | --- | --- |
| *ABL1* | chr9 | 133,710,641 | 133,763,062 | NM_005157.6 |
| *BCR* | chr22 | 23,522,696 | 23,660,224 | NM_004327.4 |
| *CBFA2T3* | chr16 | 88,941,266 | 89,043,615 | NM_005187.6 |
| *CBFB* | chr16 | 67,063,052 | 67,134,961 | NM_022845.3 |
| *CREBBP* | chr16 | 3,775,055 | 3,930,714 | NM_004380.3 |
| *DEK* | chr6 | 18,224,091 | 18,264,761 | NM_003472.4 |
| *GLIS2* | chr16 | 4,364,762 | 4,389,596 | NM_001318918.2 |
| *KDM5A* | chr12 | 389,223 | 498,486 | NM_001042603.3 |
| *KMT2A* | chr11 | 118,307,207 | 118,397,547 | NM_001197104.1 |
| *MLLT10* | chr10 | 21,823,161 | 22,032,559 | NM_001195626.3 |
| *MLLT3* | chr9 | 20,341,667 | 20,622,498 | NM_004529.4 |
| *KAT6A* | chr8 | 41,786,997 | 41,909,505 | NM_006766.5 |
| *MYH11* | chr16 | 15,796,992 | 15,950,885 | NM_002474.3 |
| *NSD1* | chr5 | 176,560,774 | 176,727,214 | NM_022455.4 |
| *NUP214* | chr9 | 134,000,973 | 134,110,050 | NM_005085.4 |
| *NUP98* | chr11 | 3,696,249 | 3,818,784 | NM_001365125.5 |
| *PICALM* | chr11 | 85,668,218 | 85,780,126 | NM_007166.4 |
| *PML* | chr15 | 74,287,057 | 74,340,168 | NM_033238.3 |
| *RARA* | chr17 | 38,465,432 | 38,513,895 | NM_000964.4 |
| *RUNX1* | chr21 | 36,160,098 | 36,421,599 | NM_001754.4 |
| *RUNX1T1* | chr8 | 92,967,195 | 93,107,882 | NM_001198679.3 |
| *FIP1L1* | chr4 | 54,243,808 | 54,327,029 | NM_001376744.1 |
| *PDGFRA* | chr4 | 55,095,460 | 55,164,412 | NM_006206.4 |
| *ETV6* | chr12 | 11,802,608 | 12,048,311 | NM_001987.4 |
| *FGFR1* | chr8 | 38,268,661 | 38,326,153 | NM_023110.2 |
| *FLT3* | chr13 | 28,577,411 | 28,674,713 | NM_004119.2 |
| *JAK2* | chr9 | 4,985,272 | 5,129,944 | NM_004972.3 |
| *NTRK3* | chr15 | 88,402,982 | 88,799,970 | NM_001012338.3 |
| *PCM1* | chr8 | 17,780,497 | 17,887,457 | NM_001352632.1 |
| *PDGFRB* | chr5 | 149,493,402 | 149,535,408 | NM_002609.4 |
| *ZMYM2* | chr13 | 20,533,164 | 20,663,255 | NM_001353162.3 |

## Table S2: ddPCR assay parameters for DNA-based SNVs and fusion genes

ddPCR assay parameters for DNA-based SNVs and fusion genes, showing molecular target, annealing temperature, Fractional Abundance (FA) or ratio at diagnosis, and limit of detection (LoD).

Abbreviations: ddPCR, droplet digital PCR; SNV, Single Nucleotide Variant; FA, Fractional Abundance; LoD: limit of detection.

| No | Molecular target | Annealing temperature (C°) | FA/Ratio diagnosis (%) | LoD |
| --- | --- | --- | --- | --- |
| 1 | *RUNX1* | 50 | 49 | 1.71 × 10⁻⁴ |
|  | *PHF6* | 54 | 48 | 3.90 × 10⁻⁴ |
| 2 | *DEK::NUP214* | 60 | 47 | 3.00 × 10⁻^5^ |
| 3 | *CBFB::MYH11* | 52 | 45 | 5.10 × 10⁻^5^ |
| 4 | *KMT2A::MLLT3* | 52 | 48 | 3.00 × 10⁻^5^ |
| 5 | *UBTF* | 52 | 39 | 3.00 × 10⁻^5^ |
| 6 | *KMT2A::MLLT10* | 52 | 41 | 5.10 × 10⁻^5^ |
| 7 | *ETV6::SYK* | 52 | 46 | 3.00 × 10⁻^5^ |
| 8 | *RUNX1::RUNXT1* | 52 | 44 | 3.00 × 10⁻^5^ |

## Table S3: ddPCR assay parameters for RNA-based mutation and fusion transcripts

ddPCR assay parameters for RNA-based mutation and fusion transcripts, showing molecular target, annealing temperature, ratio at diagnosis, and limit of detection (LOD).

Abbreviations: LoD: limit of detection.

| No | Molecular target | Annealing temperature (C°) | Ratio diagnosis (%) | LoD |
| --- | --- | --- | --- | --- |
| 2 | *DEK::NUP214* | 58 | 206 | 1.00 × 10⁻⁴ |
| 3 | *CBFB::MYH11* | 60 | 77.3 | 1.00 × 10⁻^5^ |
| 6 | *KMT2A::MLLT10* | 58 | 35.4 | 1.00 × 10⁻⁴ |
| 7 | *ETV6::SYK* | 62 | 45.1 | 3.57 × 10⁻^3^ |
| 8 | *RUNX1::RUNXT1* | 60 | 458 | 1.00 × 10⁻^5^ |
| 9 | *NPM1* | 55 | 854 | 1.00 × 10⁻⁴ |

## Table S4: Treatment received prior to OTC and disease evaluation after treatment

DNR, Daunorubicin; GO, Gemtuzumab ozogamicin; G-CSF, Granulocyte Colony-Stimulating Factor; CR, complete remission; OTC, Ovarian Tissue Cryopreservation; Y, yes; N, no

| **No** | **First-line treatment prior to OTC** | **Disease evaluation after induction** | **Relapse prior to OTC** | **Treatment for salvage/relapse prior to OTC** | **Time from CR to OTC (days)** |
| --- | --- | --- | --- | --- | --- |
| 1 | *Induction:* DNR 250 mg/m², Cytarabine 2.5 g/m²  *Consolidation:* Cytarabine 18 g/m² (1 course) | CR | N | - | 4 |
| 2 | *Induction:* DNR 270 mg/m², Cytarabine 1.4 g/m² *Consolidation:* Cytarabine 18 g/m²/course (2 courses) | CR | N | - | 8 |
| 3 | *Induction:* DNR 180 mg/m², Cytarabine 1.4 g/m², GO 6 mg/m² *Consolidation:* Cytarabine 18 g/m²/course (3 courses) | CR | Y | Fludarabine,  Cytarabine,  G-CSF, Idarubicin | 3 |
| 4 | *Induction:* DNR 250 mg/m², Cytarabine 2.5 g/m² Consolidation: Cytarabine 18 g/m²/course (2 courses) | CR | N | - | 60 |
| 5 | Induction: DNR 180 mg/m², Cytarabine 1.4 g/m², Midostaurin 1.4 g/m² | Refractory disease | N | First salvage treatment: Cytarabine, Gilteritinib Second salvage treatment: Azacitidine, Venetoclax | 53 |
| 6 | Induction: DNR 270 mg/m², Cytarabine 1.4 g/m², Midostaurin 1.4 g/m² Consolidation: Cytarabine 9 g/m²/course, Midostaurin 1.4 g/m²/course, (2 courses) | CR | N | - | 3 |
| 7 | Induction: Idarubicin 36 mg/m², Cytarabine 16 g/m² Consolidation: Cytarabine 18 g/m²/course (2 courses) | CR | N | - | 29 |
| 8 | Induction: DNR 180 mg/m², Cytarabine 1.4 g/m², GO 9 mg/m² Consolidation: Cytarabine 18 g/m² /course (3 courses) | CR | Y | Clofarabine, Cytarabine, Idarubicin | 13 |
| 9 | Induction: Cytarabine 1.4 g/m², Mitoxantrone 60 mg/m² Consolidation 1: Cytarabine 18 g/m², Amsacrine 300 mg/m² Consolidation 2: Cytarabine 800 mg/m², Etoposide 400 mg/m², DNR 160 mg/m² Consolidation 3: Cytarabine 24 g/m², L-Asparaginase 12,000 U/m² | CR | Y | Fludarabine,  Cytarabine,  G-CSF, Idarubicin, GO | 35 |
